# Physiological Responses and Performance during an Integrated High-Intensity Interval Aerobic and Power Training Protocol

**DOI:** 10.3390/sports12030076

**Published:** 2024-03-07

**Authors:** Ilias Iason Psarras, Gregory C. Bogdanis

**Affiliations:** School of Physical Education and Sports Science, National and Kapodistrian University of Athens, 17237 Athens, Greece; ipsarras@phed.uoa.gr

**Keywords:** high-intensity endurance exercise, plyometric training, neuromuscular performance, VO_2max_

## Abstract

This study compared the acute physiological responses and performance changes during an integrated high-intensity interval aerobic and power protocol. Sixteen moderately trained athletes (age: 20.1 ± 2.2 years, body height: 180.0 ± 6.5 cm, body mass: 75.7 ± 6.4 kg, VO_2max_: 55.8 ± 4.3 mL/kg/min) performed a 2 × 6 min interval training protocol with 2 min passive recovery between sets on two different occasions in random and counterbalanced order. Each 6 min set included repeated periods of 15 s exercise interspersed with 15 s passive rest. On one occasion (RUN), all exercise periods included running at 100% of maximal aerobic speed, while on the other occasion an integrated protocol was used (INT) in which each of the two 6 min sets included 4 × 1.5 min periods of running exercise at 100% of maximal aerobic speed in combination with jumping (i.e., 2 × 15 running with 15 s rest and 1 × 15 s drop jumping with 15 s rest). Time spent above 85% HR_max_ was two-fold higher in INT compared to RUN (8.5 ± 3.6 vs. 4.3 ± 3.9 min, respectively, *p* = 0.0014). Interestingly, heart rate increased above 95% HR_max_ only in INT and almost no time was spent above 95% HR_max_ in RUN (1.4 ± 1.9 vs. 0.1 ± 0.2 min, respectively, *p* = 0.008). Blood lactate concentration at the end of the second set of INT was higher than RUN (7.3 ± 3.2 vs. 4.6 ± 2.7 mmol/L, *p* = 0.002). Countermovement jump was higher in INT after the end of second set by 6.4% (*p* = 0.04), 6.7% (*p* = 0.04), 7.8% (*p* < 0.01) and 7.3% (*p* < 0.001), at 2, 6 and 8 min after set 2. In conclusion, the comparison between INT and RUN shows that INT not only elicits higher physiological and metabolic responses, but also acutely enhances neuromuscular performance for at least 8 min after the end of exercise. The integrated running/jumping high-intensity interval exercise approach could be a very useful and time efficient method for strength and conditioning coaches, especially in team sports, in which the time available for the improvement of physical parameters is limited.

## 1. Introduction

Several team sports, such as soccer and handball, require a combination of high cardiopulmonary endurance and muscle power, and thus training must target both these qualities in the limited time that is available for physical conditioning [1]. High-intensity interval exercise (HIIE) is a time-efficient method to improve aerobic fitness [2,3,4,5]. Most HIIE studies involved running, cycling or rowing exercise at intensities of around 80–100% VO_2max_ [5,6,7,8]. However, HIIE may be further divided into short- or long-type interval exercise [2]. Short intervals consist of repeated work bouts of 10–60 s at intensities ranging from 100 to 120% VO_2max_. Long intervals include repeated work bouts of >60 s duration at intensities ranging from the velocity corresponding to lactate threshold or to critical power up to 100% VO_2max_ [2]. Altering the duration of work, rest or intensity modifies the physiological, metabolic and neuromuscular responses to this type of exercise [3,4]. Interestingly, repeated jumping, instead of running, has been proposed as an effective HIIE modality to improve cardiorespiratory parameters [9,10]. In a recent study [10], an 11 min bout of repeated jumping (15 s work and 15 s rest) with nine drop jumps per 15 s of work, was shown to induce similar oxygen uptake responses compared with an equal duration and structure high-intensity running protocol (at 120% vVO_2max_). The time spent at VO_2max_ in that study was identical in the two protocols (141 s ± 151 s vs. 145 s ± 76 s) (*p* = 0.92). The effectiveness of repeated jumping in inducing considerable cardiorespiratory responses has also been demonstrated in another study [11], where eight sets of 10 drop jumps, separated by 3 min of passive recovery, elevated oxygen uptake up to 83% of VO_2max_. Thus, it seems that high frequency jumping exercise with minimized rest periods between jumps may be adequate to increase metabolic demands, elevate oxygen uptake and increase the time spent above 90% of VO_2max_, which is considered to be a key factor for increasing central and peripheral responses related to improved VO_2max_ and endurance performance [2,4]. Several studies have suggested that training at intensities close to VO_2max_ (i.e., 90–100% VO_2max_) is the most effective for enhancing VO_2max_ [12] Consequently, there is an interest in prescribing training protocols that allow the longest time near VO_2max_. Since VO_2_ and heart rate (HR) are linearly related [2], relative HR (i.e., %HR_max_) may be used as a surrogate to % VO_2max_, because it is easily measured in a continuous fashion during exercise with minimal equipment. Indeed, several studies have prescribed HR-based training protocols (i.e., running at 90–95% HR_max_) and found significant increases in VO_2max_ [2,4]. HIIE protocols have been found to be more effective than continuous protocols for increasing time spent above 90% VO_2max_ [13].

On the other hand, the use of plyometric exercises such as repeated jumps in a training session is also an effective way to improve the muscle power of the lower limbs, and thus jumping height, sprinting speed, change of direction and agility [14,15,16,17]. So far, all studies using repeated jumps to induce high cardiorespiratory load have used a large number of repetitions (150–200 jumps) performed either over a long duration (from 11 min to 25 min) [11,16,17] or with very short or no rest intervals, which may cause excessive fatigue [9]. Also, many jumps induce high neuromuscular and joint load, and thus jumping exercise protocols should be carefully prescribed to minimize fatigue and maximize cardiorespiratory responses. Evidence from a recent study [11] suggests that a rest period of 15 s between bouts of jumping results in a good balance between fatigue and cardiorespiratory responses, compared to longer rest durations. Furthermore, a jumping frequency of 0.6 jumps per second, i.e., nine jumps in 15 s, is required to attain high cardiorespiratory responses, albeit with considerable fatigue, as evidenced by evoked potentiated quadriceps twitch measurements pre- and post-exercise [10].

In an attempt to explore whether the benefits of both high-intensity running and lower limb power training can be gained during a single protocol, the purpose of the present study was to examine the physiological responses (i.e., heart rate, blood lactate concentration) and neuromuscular performance during and following a HIIE session that integrated jumping and running exercises, with an optimized structure based on the existing evidence [11,16,17]. We hypothesized that the acute physiological responses would be the same during a running and an integrated running/jumping protocol, but lower limb power would be enhanced, rather than decreased, in the integrated running/jumping HIIE protocol.

## 2. Materials and Methods

### 2.1. Participants

An a priori power analysis using repeated within-factors analysis of variance (G-Power software, v. 3.1.9.2, Universität Kiel, Kiel, Germany) indicated that a minimum sample size of 10 participants would be needed to detect a moderate effect size of 0.5, based on a power of 0.80, alpha of 0.05 and correlation coefficient of 0.5 between repeated measures. Sixteen male recreationally trained athletes (age: 20.1 ± 2.2 years, body height: 180.0 ± 6.5 cm, body mass: 75.7 ± 6.4 kg, VO_2max_: 55.8 ± 4.3 mL/kg/min, vVO_2max_: 15.4 ± 1.3 km/h) volunteered to take part in this study. Participants were amateur games players who were thoroughly familiarized with running-based HIIT formats. Before participation, all athletes signed an informed consent form and completed a health history questionnaire. None of them had any injuries for at least one year before the experimental sessions. Also, participants were non-smokers and did not take any medications or supplements which could affect their performance or metabolism. Players were instructed to maintain the same diet for 24 h and to avoid intense workouts for at least 48 h prior to each session. All procedures were in accordance with the Declaration of Helsinki and approved by the local university ethics committee (n.1467/11-01-23, approval date 11 January 2023).

### 2.2. Procedures

Participants took part in one familiarization session, one preliminary measurements session and two main sessions 3–7 days apart. Before every familiarization, preliminary or main experimental session, a standardized warm up was performed, including 5 min running at 65–75% of maximum heart rate and 5 min of dynamic stretching.

The familiarization session included a standardized warm-up and drop jumps from different heights. Also, part (10 min) of the multistage aerobic shuttle run test was performed in this session. In the preliminary session, the drop jump height used in the integrated session was determined. Furthermore, VO_2max_ and velocity at VO_2max_ (vVO_2max_) were estimated using the multistage shuttle run test [18] and vVO_2max_ was calculated by Berthoin’s equation [19]. To determine the individual drop jump height, participants executed two jumps from each of four different heights (30 cm, 40 cm, 50 cm and 60 cm) with 1 min passive recovery between jumps and 3 min between heights with random and counterbalanced order. Jump height was calculated from flight time, using a recent mobile app named “My Jump 2”. This application is compatible with iPhone devices (Apple, Inc., Cupertino, CA, USA) which have relatively high video sampling rates (240 fps) and have been shown to provide valid and reliable data during fast stretch shortening cycle (SSC) exercises such as drop jumps and sprints, and during slow SSC exercises such as the counter movement jump (CMJ) [20,21,22,23,24]. Power output was calculated using the Sayers’s equation [25]. The jump height eliciting the highest power output was chosen as the individual optimum and was used during the main sessions. Following 15 min of rest after the drop jump test, participants performed a 20 m shuttle run test to evaluate VO_2max_ and vVO_2max_ according to Leger’s protocol [18].

Two main sessions were performed in a random and counterbalanced order. They both included two sets of 6 min (15 s exercise bouts interspersed with 15 s intervals of passive recovery) with a 2 min passive rest in between sets. On one occasion, all 15 s exercise bouts included running at high intensity (100% vVO_2max_), while on the other occasion the 15 s exercise bouts included running at the same intensity (100% vVO_2max_), as well as repeated jumping from the predetermined individual drop jump height. Details of this protocol are presented below. The recovery time between sessions was 3–7 days. Heart rate (HR) was measured during each main session and for 1 min into recovery after the end of the exercise, since it is an appropriate index of exercise intensity and is linearly related to oxygen consumption [2,4]. Blood lactate (BLa) was measured before and after the first and second set of each condition. Neuromuscular performance was assessed via the CMJ with the hands on the hips, before and after the first and second set, and also 2, 4, 6, 8 and 10 min after the end of each session.

This study has certain limitations. To measure the level of increase in aerobic metabolism, it would have been useful to record and compare VO_2_ during each protocol. Of course, this would require a portable device which was not available at the time of the study. Also, it would have been interesting to apply this protocol to a target population, such as in football players, who may use it during their weekly microcycle. Finally, the mechanisms of fatigue during and after the experimental sessions could have been examined with a more precise method, e.g., electric nerve stimulation or transcranial magnetic stimulation, instead of an indirect indicator such as CMJ height.

### 2.3. Running and Integrated Sessions

Prior to each main test, a standardized warm-up was undertaken, including 5 min jogging at 65–75% HR_max_ followed by 5 min dynamic stretching. Three minutes after the end of the standardized warm-up, BLa and CMJ were measured (within one minute), and the main session started one minute later (i.e., 4 min after the end of the warm-up).

**Figure 1:**
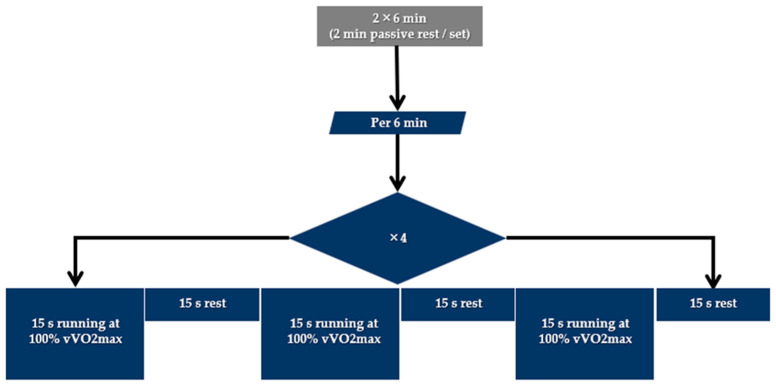
Structure of the running (RUN) session.

The running session (RUN) included two 6 min sets of high-intensity running exercise, as illustrated in Figure 1. The participants had to complete 12 repetitions of running for 15 s at 100% of vVO_2max_ interspersed with 15 s intervals of passive recovery [26,27]. The recovery interval between sets was 2 min.

**Figure 2:**
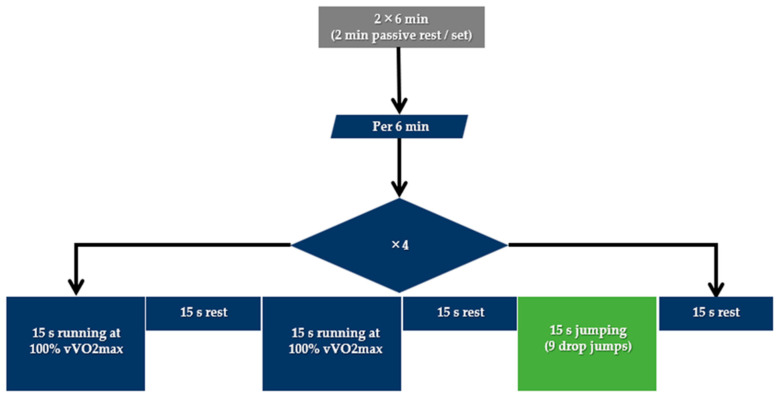
Structure of the integrated (INT) running and jumping session.

**Figure 3:**
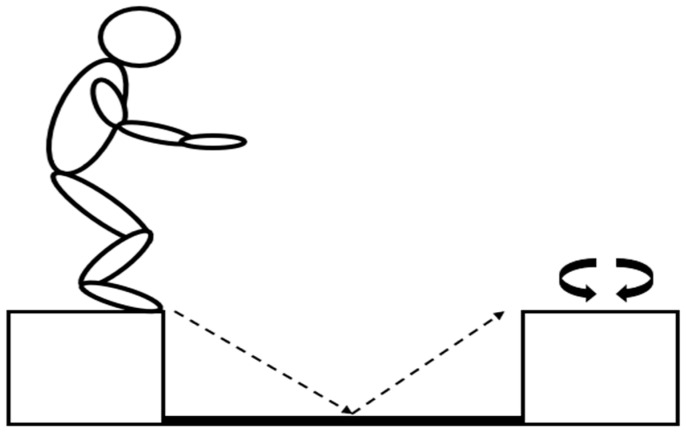
Schematic illustration of the jumping part of the integrated protocol.

The integrated (INT) session included a combination of high-intensity running and jumping exercises of 2 × 6 min, as illustrated in Figure 2. During each 6 min set, the participants had to complete 4 consecutive rounds of 1.5 min, each including 2 bouts of 15 s running at 100% vVO_2max_ and one 15 s bout of jumping (9 drop jumps) from the predetermined height, all interspersed with 15 s of passive recovery. The drop jumping exercise was executed using two boxes of appropriate height, as shown in Figure 3. The distance between the two boxes was 1 m and a metronome was used to determine drop jump rate.

Participants had to jump from one box to the other as fast as possible, minimizing the ground contact time, and then turn their body on the box in the opposite direction for the next jump. The total number of jumps in each session was 72. The recovery interval between sets was 2 min.

### 2.4. Physiological Measurements

HR was measured continuously every 1 s using a Polar H10 Polar monitor (Polar Electro Oy, Kempele, Finland). HR was also expressed as a percentage of maximum heart rate (HR_max_) measured during the shuttle run test to exhaustion. The following parameters were obtained during the two experimental protocols: peak and mean HR in both sets, time spent between 85 and 95% HR_max_ and time spent >90% and >95% HR_max_ [10]. Also, the drop in HR in the first min after the end of the second 6 min set was calculated.

Blood lactate concertation was measured using a portable device (Lactate Scout 4 LS, SensLab GmbH, Leipzig, Germany), before and after the first and second set of each condition.

### 2.5. Statistical Analysis

Data analysis was performed using IBM SPSS Statistics Version 26. Descriptive statistics were calculated (mean values and standard deviations). Two-way analysis of variance (ANOVA) with repeated measures on both factors was conducted and when significant differences were found, multiple comparisons analyses were performed using Tukey’s HSD test. Cohen’s d was used for the calculation of effect sizes (ES). Statistical significance was set at *p* < 0.05.

## 3. Results

### 3.1. Heart Rate

**Figure 4:**
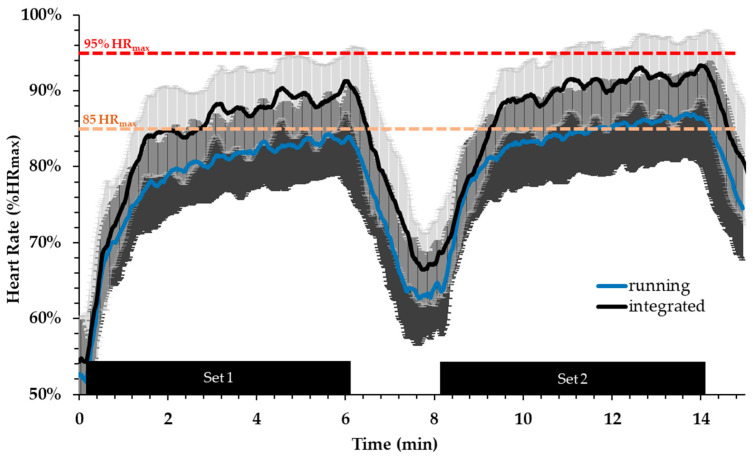
Heart rate during the integrated aerobic and power session and running-only session. Orange line indicates 85% of HR_max_ and red line indicates 95% of HR_max_.

HR was higher during INT compared with RUN (F = 2.865, *p* < 0.001) (Figure 4). Specifically, peak HR was 7.6% higher in the first set and 6.8% higher in the second set in the INT compared to the RUN condition, respectively (first set: 177 ± 17 vs. 163 ± 15 bpm, *p* < 0.001, ES = 0.88; second set: 181 ± 16 vs. 169 ± 14 bpm, *p* < 0.001, ES = 0.8). Mean heart rate (HR) was 6.6% higher during the first set and 6.4% higher during the second set in the INT compared to the RUN condition, respectively (first set: 161 ± 17 vs. 151 ± 14 bpm, *p* < 0.001, ES = 0.67; second set: 169 ± 13 vs. 160 ± 10 bpm, *p* < 0.001, ES = 0.82). Moreover, the average HR during the recovery interval after the first set was 7.4% higher in INT compared to RUN (149 ± 16 vs. 138 ± 14 bpm, *p* < 0.001, ES = 0.73). Time spent above 85% HR_max_ was two-fold higher in INT compared to RUN (8.5 ± 3.6 vs. 4.3 ± 3.9 min, respectively, *p* = 0.0014, ES = 1.1). Time spent above 90% HR_max_ was more than 4-fold higher in INT than RUN (5.0 ± 4.0 vs. 1.2 ± 2.3 min, respectively, *p* = 0.0004, ES = 1.08). The larger part of time above 85% HR_max_ was spent between 85% and 95% HR_max_ (7.1 ± 2.9 vs. 4.2 ± 3.9 min, for INT and RUN, respectively, *p* = 0.023, ES = 0.85). Interestingly, heart rate increased above 95% HR_max_ only in INT and almost no time was spent at that HR zone in RUN (1.4 ± 1.9 vs. 0.1 ± 0.2 min, respectively, *p* = 0.008, ES = 0.76). 

### 3.2. Blood Lactate Concentration

**Figure 5:**
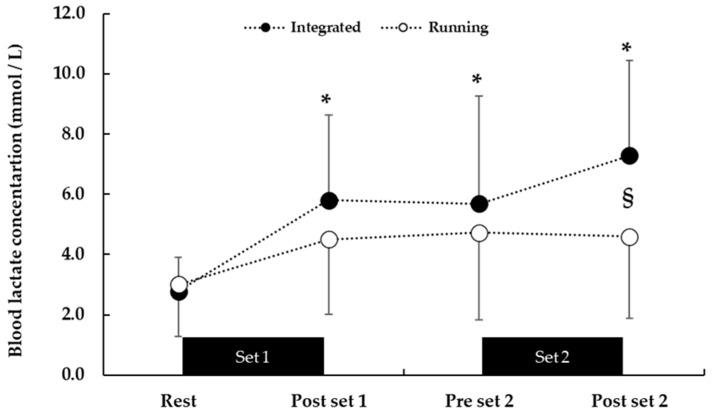
Blood lactate concentration during the integrated aerobic and power session (black) and the running only session (white). Time corresponds to that after the start of the first set (see Figure 5). * *p* < 0.001 from rest, § *p* < 0.05 between conditions.

BLa concentration was higher at INT compared to RUN both during and after HIIE (F = 3.82, *p* = 0.01) (Figure 5). BLa was similar in both conditions before the beginning of the sessions (2.8 mmol/L for the INT vs. 3.0 mmol/L for the RUN). After the first set, BLa increased similarly in both conditions (5.8 ± 2.8 and 4.5 ± 2.5 mmol/L; in INT and RUN, respectively, *p* < 0.001, ES = 0.5), and remained unchanged in both conditions before the second set (INT: 5.7 ± 3.6 and RUN: 4.7 ± 2.9 mmol/L). After the end of the second set, BLa concertation further increased in INT and was higher than RUN (7.3 ± 3.2 vs. 4.6 ± 2.7 mmol/L, *p* < 0.05, ES = 0.9).

### 3.3. Neuromuscular Performance

**Figure 6:**
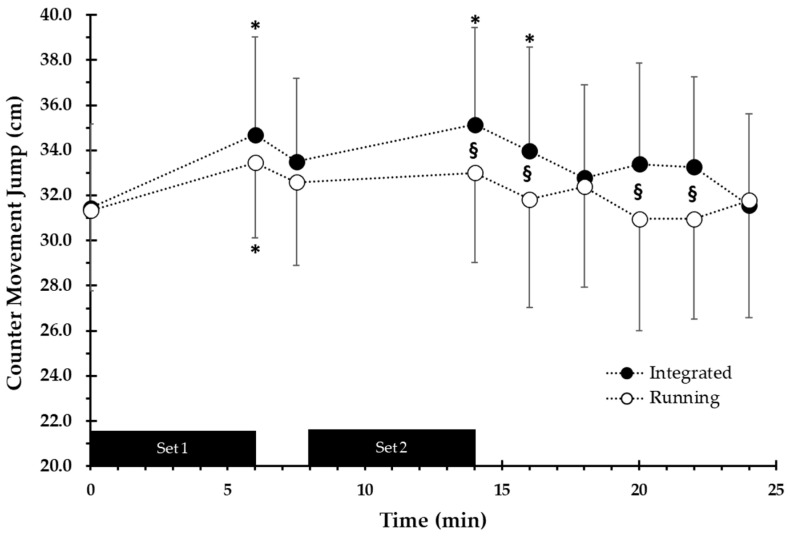
Countermovement jump performance during the integrated aerobic/power session and the running session. * *p* < 0.001 from baseline, § *p* < 0.05 between conditions.

Neuromuscular performance, as assessed by the CMJ, was greater at INT compared to RUN (F = 2.88, *p* = 0.005). CMJ was similar in both conditions before the first set (INT: 31.4 ± 3.7 cm and RUN: 31.3 ± 3.6 cm). After the end of the first set, CMJ performance increased by 10.3% and 6.8% in INT (34.7 ± 4.3 cm, *p* < 0.001, ES = 0.82) and RUN (33.5 ± 3.3 cm, *p* = 0.045, ES = 0.66), respectively. After the end of the second set, CMJ height was 11.8% higher compared with baseline only in INT (35.1 ± 4.3, *p* < 0.001, ES = 0.9). CMJ performance in INT remained 6.1 to 7.3% higher (*p* = 0.041 to 0.007) than RUN during the first 8 min of recovery after the second set (Figure 6).

## 4. Discussion

The purpose of the present study was to investigate the acute physiological effects of an integrated HIIE protocol in which running and jumping was combined (INT), and to compare it with a running-only HIIE protocol of similar structure and duration (RUN). An important point when designing HIIE programs is the appropriate adjustment of the intensity and duration of the exercise and recovery parts, as well as the type of exercise (running, cycling, rowing, etc.) [2]. However, most studies to date have employed linear running or cycling [7,8,26,27,28], with only a limited number of studies examining alternative exercise modes (e.g., repeated jumping) [11,12]. In the present study, we combined running and jumping exercises into a commonly used HIIE format (15 s exercise, 15 s rest) used for aerobic training in several team or intermittent sports [11,12,29]. By doing this, we wanted to explore whether the benefits of both high-intensity running and jumping exercises can be gained in the same training protocol.

One main finding of the present study was that HR increased to higher levels in the INT session compared with the RUN session, and this suggests that this integrated HIIE format is more effective than the running-only format regarding cardiorespiratory loading. Therefore, the combination of drop jumps and running in a short rest interval format (15 s work, 15 s rest) seems to be superior to running only, in terms of aerobic metabolism stimulation. Previous studies used either running-only or jumping-only protocols of HIIE and have shown that both are effective in raising VO_2_ and HR to comparable levels [11,12]. For example, Ducronq et al. [10] compared a HIIE running protocol (15 s running at 120% vVO_2max_ and 15 s passive recovery) with a drop-jump-only protocol of equal duration and structure including nine jumps per 15 s. The findings showed similar oxygen uptake responses in the two protocols (time spent at VO_2max_: 141 ± 151 vs. 145 ± 76 s in the running and jumping protocols, respectively). The effectiveness of jumping in raising aerobic metabolism has also been demonstrated in the past [9], where a jumping-only protocol was examined using different combinations of work and rest durations. In that study, the shorter duration of recovery resulted in higher HR levels compared with the longer recovery intervals, and this increased the time spent above 80% and 90% of HR_max_ and VO_2max_. The benefits of this short recovery duration between short bouts of exercise are also evident in other studies, in which longer recovery durations (from 30 s to 3 min) resulted in reduced HR and VO_2_ responses [11,13,29]. 

The selection of a moderate number of jumps in the present study (i.e., a total of 72 jumps) in combination with running (INT) was based on a critical examination of the previous literature [11,12]. In accordance with those studies, a reduction in the number of jumps per set from nine to seven would have caused a dramatic decrease in the time spent near VO_2max_and HR_max_ [10]. It is well known that the time spent above 90% of HR_max_ is considered to be crucial for the effectiveness of an aerobic program, with the guidelines suggesting that at least 5 to 10 min above 90% HR_max_ is necessary to promote aerobic adaptations such as increases in VO_2max_ and lactate threshold [4]. In the present study, the time spent above 90% HR_max_ was more than four-fold higher in INT than RUN (5.0 ± 4.0 vs. 1.2 ± 2.3 min, respectively, *p* = 0.0004), while heart rate increased above 95% HR_max_ only in INT (1.4 ± 1.9 vs. 0.1 ± 0.2 min, respectively, *p* = 0.008). These results suggest that the HIIE 15-15 s running-only protocol at 100% vVO_2max_ may not provide the optimal stimulus for enhancing VO_2max_, and a higher intensity should be chosen for this purpose [21]. In contrast, the addition of jumping bouts in between running elevated HR at higher levels, with the participants spending more than four-fold longer time above 90% HR_max_ compared to RUN.

Another important finding of the present study was that neuromuscular performance, as assessed by CMJ, was higher in INT compared with the RUN immediately after the end of the session and for 8 min during recovery. This difference between INT and RUN was 6–7% (see Figure 6) and suggests that neuromuscular performance was enhanced rather than reduced due to fatigue following the integrated running/jumping protocol. This is in contrast with the findings of another study [12], which reported significant decreases in evoked potentiated quadriceps twitch after a jumping protocol using either 7 or 9 jumps per 15 s, with 15 s rest, executed for 11 min. This neuromuscular fatigue observed after 11 min of high-intensity intermittent jumping is possibly related to the high number of jumps performed in the two protocols of that study [12], i.e., 154 jumps in total when performing 7 jumps per bout and 198 jumps when performing 9 drop jumps per 15 s bout. In the present study, we have chosen to use a relatively low total volume of jumps, which was only 35–50% of the number of jumps employed in previous studies (i.e., 72 vs. 150–200 jumps) [11,12,13]. This reduced volume of plyometric exercises possibly had a positive effect on the balance between muscle fatigue and activation, resulting in improved jump performance. Previous studies have shown that running alone does not reduce neuromuscular performance during protocols of HIIE performed at 100% of vVO_2max_, even if a longer work interval duration is used (e.g., 60 s or 120 s of work with equal rest) [30]. In the present study, the increased CMJ performance observed at the end and for 8 min following the integrated protocol may be explained by the phenomenon of post activation performance enhancement (PAPE) [31,32], possibly induced by the relatively low number of plyometric jumps performed. Although the mechanisms of PAPE have not been investigated in the present study, it is possible that the low volume of repeated jumps in combination with the low volume of the endurance exercise resulted in a better balance between neural activation and fatigue [27]. Several studies have highlighted that inappropriate handling of exercise volume, intensity and rest periods may cause fatigue. For example, executing 100 drop jumps from 40 cm (10 sets of 10 repetitions) immediately prior to a 4 km time trial caused an acute reduction in neuromuscular function (i.e., decrease in potentiated quadriceps twitch-force and voluntary activation) compared with a control trial [33]. In the present study, a total of 72 drop jumps were performed, but the structure of the INT protocol resulted in improved rather than decreased CMJ performance during and after the INT session. Interestingly, a study investigating the effects of drop jumps with different volumes (one, two or three sets of three repetitions) and intra-repetition recovery periods (15 s, 4 min, 8 min and 12 min) on sprint performance showed that the use of one set of three drop jumps with 15 s recovery between jumps acutely increased the 5 m and 10 m sprint performance by 3.38% and 2.07%, respectively [34]. Our finding, that the potentiation of CMJ performance lasts for 8 min after the end of the protocol, is in agreement with previous findings showing PAPE 4–12 min after the conditioning exercise [32].

Blood lactate concentration was higher at the end of the integrated protocol compared with the running-only protocol (see Figure 5), possibly indicating higher glycolytic demands when combining running with jumping exercise in this HIIE protocol. Similar lactate concentrations have been observed previously during jumping-only protocols, albeit with a much higher number of jumps (9 sets of 20 jumps with 15 s recovery in between in 8 min) [9]. From a practical viewpoint, an increased glycolytic contribution, reflected by higher blood lactate values in INT, may enhance adaptations related to reduced muscle fatigue during intense intermittent exercise, such as increases in glycolytic and oxidative enzyme activity, and a higher expression of membrane transport proteins involved in pH regulation and muscle Na^+^, K^+^ pump α-subunits [35]. The fact that despite a considerable level of metabolic stress CMJ was enhanced and heart rate responses were kept high in the present study suggests that this combination of running and jumping may be most appropriate for both cardiorespiratory and neuromuscular adaptations. 

In conclusion, this study showed that combining running and jumping in a carefully designed HIIE program may acutely increase the HR responses and enhance neuromuscular performance during and after HIIE. It is possible that a long-term application of the integrated protocol may elicit higher aerobic and leg power improvements than a running-only protocol. Future studies should investigate chronic adaptations of similar programs as well as the time course of these adaptations. The integrated running/jumping HIIE approach could be a very useful and time efficient method for strength and conditioning coaches, especially in team sports in which the time available for the improvement of physical parameters is limited. The INT protocol could be used in strength and conditioning programs either during off-season for the maintenance of fitness capacities or in the pre-season phase for fitness development.

## Data Availability

The data presented in this study are available from the corresponding author upon request. The data are not publicly available due to privacy restrictions.
